# [(4-Dimethyl­amino-2-methyl-5-phenyl­furan-3-yl)meth­yl]diethyl­methyl­aza­nium iodide

**DOI:** 10.1107/S1600536811037743

**Published:** 2011-09-20

**Authors:** Armen Ayvazyan

**Affiliations:** a Molecule Structure Research Center, Scientific Technological Center of Organic and Pharmaceutical Chemistry, National Academy of Sciences, Republic of Armenia, Azatutyan Ave. 26, Yerevan 0014, Armenia

## Abstract

In the title compound, C_19_H_29_N_2_O^+^·I^−^, the dihedral angle between the mean planes of the essentially planar furan (r.m.s. deviation = 0.007 Å) and phenyl rings is 48.4 (1)°. In the crystal, cations and anions are arranged in layers lying parallel to (100).

## Related literature

For the biological activities of furan derivatives, see: Chen *et al.* (2006 ▶); Meotti *et al.* (2003 ▶); Kazuo *et al.* (2001 ▶). For details of the synthesis, see: Manukyan *et al.* (2007 ▶). For standard bond lengths, see: Allen *et al.* (1987 ▶). 
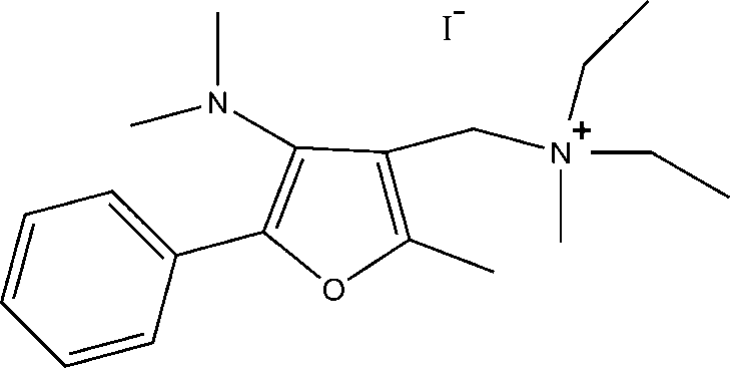


## Experimental

### 

#### Crystal data

C_19_H_29_N_2_O^+^·I^−^
*M* *_r_* = 428.34
Monoclinic, 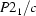

*a* = 17.905 (4) Å
*b* = 7.2458 (14) Å
*c* = 15.732 (3) Å
β = 94.84 (3)°
*V* = 2033.7 (7) Å^3^
*Z* = 4
Mo *K*α radiation
μ = 1.58 mm^−1^
*T* = 293 K
0.4 × 0.36 × 0.3 mm

#### Data collection

Enraf–Nonius CAD-4 diffractometer
Absorption correction: ψ scan (*PLATON*; Spek, 2009 ▶) *T*_min_ = 0.395, *T*_max_ = 0.430
6119 measured reflections
5911 independent reflections
4370 reflections with *I* > 2σ(*I*)
*R*_int_ = 0.013
3 standard reflections every 60 min  intensity decay: none

#### Refinement

*R*[*F* ^2^ > 2σ(*F* ^2^)] = 0.032
*wR*(*F* ^2^) = 0.081
*S* = 0.99
5911 reflections
258 parameters
H atoms treated by a mixture of independent and constrained refinement
Δρ_max_ = 0.67 e Å^−3^
Δρ_min_ = −0.55 e Å^−3^

### 

Data collection: *CAD-4 Software* (Enraf–Nonius, 1988 ▶); cell refinement: *SETANG* in *CAD-4 Software* (Enraf–Nonius, 1988 ▶); data reduction: *HELENA* (Spek, 1997 ▶); program(s) used to solve structure: *SHELXS97* (Sheldrick, 2008 ▶); program(s) used to refine structure: *SHELXL97* (Sheldrick, 2008 ▶); molecular graphics: *SHELXTL* (Sheldrick, 2008 ▶); software used to prepare material for publication: *enCIFer* (Allen *et al.*, 2004 ▶).

## Supplementary Material

Crystal structure: contains datablock(s) global, I. DOI: 10.1107/S1600536811037743/lh5334sup1.cif

Structure factors: contains datablock(s) I. DOI: 10.1107/S1600536811037743/lh5334Isup2.hkl

Supplementary material file. DOI: 10.1107/S1600536811037743/lh5334Isup3.cml

Additional supplementary materials:  crystallographic information; 3D view; checkCIF report

## Figures and Tables

**Fig. 1.**
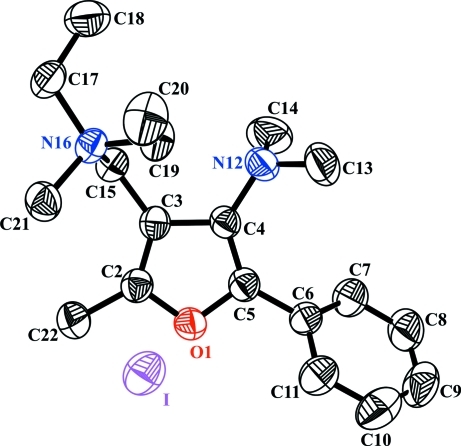
The molecular structure with displacement displacement ellipsoids drawn at the 50% probability level (H atoms are omitted for clarity).

**Fig. 2.**
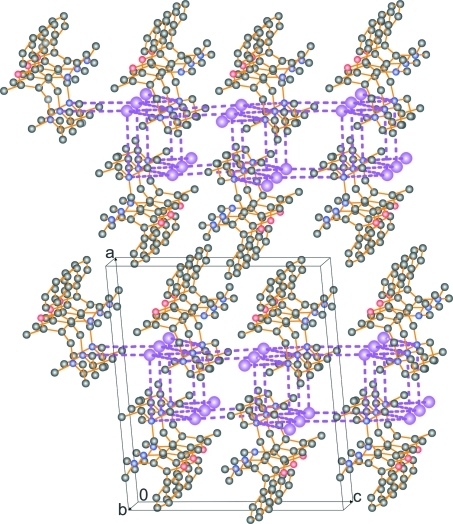
Part of the crystal structure emphasizing arrangement of cations and anions with dashed lines.

## supplementary crystallographic information

### Comment

Compounds containing furan rings are distinguished by a number of interesting biological properties. In particular, the certain representatives of this family show antibacterial, antioxidant and anti-inflammatory activities (Chen *et al.*, 2006, Meotti *et al.*, 2003, Kazuo *et al.*, 2001). Such a diversity of biological properties of these materials stimulates the interest in their structural studies. The asymmetric unit of title compound is shown in Fig.1. The molecule contains one quaternary N^+^ cation, the positive charge on which is balanced by an iodide anion. All bond lengths (Allen *et al.*, 1987) and angles in good agreement with their standard values. In the crystal, cations and anions are arranged in layers parallel to (100) with N^+^···I^-^ distances in the range 4.429 (1) Å–5.332 (1) Å (see Fig. 2).

### Experimental

The title compound was synthesized *via* Stevens rearrangement by the interaction of methyl iodide and 2-phenyl-3-dimethylamino-4-diethylamino-5-methylfuran (Manukyan *et al.*, 2007). Single crystals were grown by slow evaporation of a solution of the title compound in ethanol at room temperature.

### Refinement

H atom positions (except of those belonging to methyl groups) were located in difference Fourier maps and their positions and *U*_iso_ values were freely refined. H atoms of methyl groups were positioned geometrically and refined using a riding model with C—H = 0.96 Å and *U*_iso_(H) = 1.5U_eq_(C).

### Crystal data

**Table:**

C_19_H_29_N_2_O^+^·I^−^	*F*(000) = 872
*M**_r_* = 428.34	*D*_x_ = 1.399 Mg m^−^^3^
Monoclinic, *P*2_1_/*c*	Melting point: 353 K
Hall symbol: -P 2ybc	Mo *K*α radiation, λ = 0.71073 Å
*a* = 17.905 (4) Å	Cell parameters from 25 reflections
*b* = 7.2458 (14) Å	θ = 13.9–16.3°
*c* = 15.732 (3) Å	µ = 1.58 mm^−^^1^
β = 94.84 (3)°	*T* = 293 K
*V* = 2033.7 (7) Å^3^	Prism, red
*Z* = 4	0.4 × 0.36 × 0.3 mm

### Data collection

**Table:**

Enraf–Nonius CAD-4 diffractometer	4370 reflections with *I* > 2σ(*I*)
Radiation source: fine-focus sealed tube	*R*_int_ = 0.013
graphite	θ_max_ = 30.0°, θ_min_ = 1.1°
θ/2θ scans	*h* = −25→25
Absorption correction: ψ scan (*PLATON*; Spek, 2009)	*k* = −10→0
*T*_min_ = 0.395, *T*_max_ = 0.430	*l* = 0→22
6119 measured reflections	3 standard reflections every 60 min
5911 independent reflections	intensity decay: none

### Refinement

**Table:**

Refinement on *F*^2^	Primary atom site location: structure-invariant direct methods
Least-squares matrix: full	Secondary atom site location: difference Fourier map
*R*[*F*^2^ > 2σ(*F*^2^)] = 0.032	Hydrogen site location: mixed
*wR*(*F*^2^) = 0.081	H atoms treated by a mixture of independent and constrained refinement
*S* = 0.99	*w* = 1/[σ^2^(*F*_o_^2^) + (0.0331*P*)^2^ + 0.9333*P*] where *P* = (*F*_o_^2^ + 2*F*_c_^2^)/3
5911 reflections	(Δ/σ)_max_ = 0.002
258 parameters	Δρ_max_ = 0.67 e Å^−^^3^
0 restraints	Δρ_min_ = −0.55 e Å^−^^3^

### Special details

**Table:**

Geometry. All e.s.d.'s (except the e.s.d. in the dihedral angle between two l.s. planes) are estimated using the full covariance matrix. The cell e.s.d.'s are taken into account individually in the estimation of e.s.d.'s in distances, angles and torsion angles; correlations between e.s.d.'s in cell parameters are only used when they are defined by crystal symmetry. An approximate (isotropic) treatment of cell e.s.d.'s is used for estimating e.s.d.'s involving l.s. planes.
Refinement. Refinement of *F*^2^ against ALL reflections. The weighted *R*-factor *wR* and goodness of fit *S* are based on *F*^2^, conventional *R*-factors *R* are based on *F*, with *F* set to zero for negative *F*^2^. The threshold expression of *F*^2^ > σ(*F*^2^) is used only for calculating *R*-factors(gt) *etc*. and is not relevant to the choice of reflections for refinement. *R*-factors based on *F*^2^ are statistically about twice as large as those based on *F*, and *R*- factors based on ALL data will be even larger.

### Fractional atomic coordinates and isotropic or equivalent isotropic displacement parameters (Å^2^)

**Table:**

	*x*	*y*	*z*	*U*_iso_*/*U*_eq_	
I	0.360258 (9)	0.60153 (2)	0.329552 (12)	0.05986 (7)	
O1	0.17460 (9)	0.8773 (2)	0.27018 (10)	0.0496 (4)	
C2	0.22386 (12)	1.0199 (3)	0.26901 (15)	0.0467 (5)	
C3	0.23667 (11)	1.0608 (3)	0.18794 (14)	0.0440 (5)	
C4	0.19021 (11)	0.9379 (3)	0.13392 (14)	0.0444 (5)	
C5	0.15362 (12)	0.8279 (4)	0.18675 (14)	0.0465 (5)	
C6	0.10368 (12)	0.6681 (4)	0.17655 (15)	0.0497 (5)	
C7	0.03983 (15)	0.6718 (5)	0.1197 (2)	0.0673 (7)	
H7	0.0271 (19)	0.788 (5)	0.089 (2)	0.092 (11)*	
C8	−0.00691 (18)	0.5198 (6)	0.1117 (2)	0.0806 (10)	
H8	−0.046 (2)	0.519 (5)	0.073 (2)	0.094 (11)*	
C9	0.0084 (2)	0.3651 (5)	0.1599 (3)	0.0834 (11)	
H9	−0.020 (2)	0.259 (5)	0.154 (2)	0.091 (11)*	
C10	0.0716 (2)	0.3597 (5)	0.2157 (3)	0.0831 (10)	
H10	0.0835 (19)	0.267 (5)	0.249 (2)	0.083 (11)*	
C11	0.11910 (17)	0.5108 (4)	0.2253 (2)	0.0659 (7)	
H11	0.1584 (17)	0.506 (5)	0.2656 (19)	0.070 (9)*	
N12	0.18903 (11)	0.9401 (3)	0.04386 (13)	0.0568 (5)	
C13	0.19220 (18)	0.7624 (5)	0.00155 (19)	0.0816 (10)	
H13A	0.2260	0.6824	0.0348	0.122*	
H13B	0.2096	0.7792	−0.0540	0.122*	
H13C	0.1431	0.7083	−0.0041	0.122*	
C14	0.1351 (2)	1.0677 (6)	0.0018 (2)	0.0916 (11)	
H14A	0.0855	1.0187	0.0034	0.137*	
H14B	0.1459	1.0839	−0.0565	0.137*	
H14C	0.1384	1.1845	0.0306	0.137*	
C15	0.28935 (12)	1.2003 (3)	0.15767 (16)	0.0465 (5)	
H15A	0.2958 (15)	1.312 (4)	0.1934 (17)	0.061 (7)*	
H15B	0.2725 (13)	1.242 (3)	0.0983 (16)	0.049 (7)*	
N16	0.36958 (10)	1.1291 (2)	0.15215 (12)	0.0445 (4)	
C17	0.41598 (14)	1.2812 (4)	0.11586 (18)	0.0551 (6)	
H17A	0.4678 (15)	1.232 (4)	0.1215 (16)	0.058 (7)*	
H17B	0.4089 (17)	1.395 (4)	0.154 (2)	0.072 (9)*	
C18	0.39221 (18)	1.3347 (5)	0.02513 (19)	0.0740 (8)	
H18A	0.3949	1.2288	−0.0111	0.111*	
H18B	0.4249	1.4294	0.0071	0.111*	
H18C	0.3417	1.3800	0.0215	0.111*	
C19	0.36726 (14)	0.9537 (4)	0.09966 (19)	0.0543 (6)	
H19A	0.3403 (15)	0.988 (4)	0.0445 (18)	0.059 (7)*	
H19B	0.3352 (17)	0.867 (4)	0.1319 (18)	0.065 (8)*	
C20	0.44255 (16)	0.8662 (4)	0.0897 (2)	0.0725 (8)	
H20A	0.4770	0.9588	0.0736	0.109*	
H20B	0.4372	0.7728	0.0464	0.109*	
H20C	0.4613	0.8114	0.1428	0.109*	
C21	0.40518 (14)	1.0903 (4)	0.24027 (16)	0.0552 (6)	
H21A	0.3786	0.9921	0.2655	0.083*	
H21B	0.4032	1.1993	0.2747	0.083*	
H21C	0.4565	1.0545	0.2369	0.083*	
C22	0.25056 (17)	1.0946 (4)	0.35412 (16)	0.0636 (7)	
H22A	0.2853	1.0094	0.3826	0.095*	
H22B	0.2086	1.1118	0.3875	0.095*	
H22C	0.2750	1.2109	0.3472	0.095*	

### Atomic displacement parameters (Å^2^)

**Table:**

	*U*^11^	*U*^22^	*U*^33^	*U*^12^	*U*^13^	*U*^23^
I	0.05869 (10)	0.04681 (9)	0.07390 (12)	0.00165 (7)	0.00458 (8)	0.00206 (8)
O1	0.0473 (8)	0.0588 (10)	0.0431 (8)	−0.0070 (7)	0.0066 (6)	−0.0025 (7)
C2	0.0431 (11)	0.0487 (12)	0.0484 (12)	−0.0015 (9)	0.0054 (9)	−0.0034 (10)
C3	0.0374 (10)	0.0457 (11)	0.0484 (12)	0.0002 (8)	0.0015 (8)	−0.0017 (9)
C4	0.0362 (9)	0.0547 (13)	0.0422 (11)	−0.0021 (9)	0.0022 (8)	−0.0031 (9)
C5	0.0379 (10)	0.0556 (13)	0.0463 (12)	−0.0044 (9)	0.0044 (8)	−0.0045 (10)
C6	0.0397 (11)	0.0609 (14)	0.0496 (12)	−0.0104 (10)	0.0109 (9)	−0.0038 (11)
C7	0.0503 (14)	0.082 (2)	0.0690 (17)	−0.0184 (14)	0.0016 (12)	−0.0006 (16)
C8	0.0576 (16)	0.105 (3)	0.078 (2)	−0.0328 (18)	0.0029 (15)	−0.012 (2)
C9	0.073 (2)	0.084 (2)	0.097 (2)	−0.0362 (18)	0.0300 (18)	−0.020 (2)
C10	0.081 (2)	0.069 (2)	0.103 (3)	−0.0127 (17)	0.026 (2)	0.0092 (19)
C11	0.0575 (15)	0.0672 (18)	0.0736 (19)	−0.0084 (14)	0.0094 (14)	0.0031 (15)
N12	0.0507 (11)	0.0776 (15)	0.0419 (10)	−0.0121 (10)	0.0028 (8)	−0.0029 (10)
C13	0.082 (2)	0.106 (3)	0.0587 (16)	−0.0292 (19)	0.0181 (14)	−0.0309 (17)
C14	0.090 (2)	0.119 (3)	0.0623 (18)	−0.009 (2)	−0.0130 (16)	0.0278 (19)
C15	0.0423 (11)	0.0432 (12)	0.0536 (13)	−0.0004 (9)	0.0014 (9)	0.0028 (10)
N16	0.0387 (8)	0.0426 (10)	0.0517 (10)	−0.0046 (7)	0.0017 (7)	0.0016 (8)
C17	0.0491 (13)	0.0508 (13)	0.0653 (15)	−0.0121 (11)	0.0036 (11)	0.0034 (12)
C18	0.0786 (19)	0.077 (2)	0.0674 (18)	−0.0070 (16)	0.0140 (15)	0.0166 (16)
C19	0.0459 (12)	0.0503 (13)	0.0667 (16)	−0.0031 (10)	0.0048 (11)	−0.0124 (12)
C20	0.0537 (14)	0.0706 (19)	0.095 (2)	0.0021 (13)	0.0139 (14)	−0.0185 (16)
C21	0.0529 (12)	0.0562 (14)	0.0546 (13)	0.0000 (11)	−0.0075 (10)	0.0025 (11)
C22	0.0742 (17)	0.0670 (17)	0.0497 (13)	−0.0135 (14)	0.0061 (12)	−0.0102 (13)

### Geometric parameters (Å, °)

**Table:**

O1—C2	1.360 (3)	C14—H14B	0.9600
O1—C5	1.382 (3)	C14—H14C	0.9600
C2—C3	1.348 (3)	C15—N16	1.536 (3)
C2—C22	1.485 (3)	C15—H15A	0.99 (3)
C3—C4	1.445 (3)	C15—H15B	1.00 (2)
C3—C15	1.488 (3)	N16—C21	1.503 (3)
C4—C5	1.359 (3)	N16—C19	1.514 (3)
C4—N12	1.415 (3)	N16—C17	1.520 (3)
C5—C6	1.463 (3)	C17—C18	1.505 (4)
C6—C11	1.388 (4)	C17—H17A	0.99 (3)
C6—C7	1.391 (4)	C17—H17B	1.04 (3)
C7—C8	1.383 (4)	C18—H18A	0.9600
C7—H7	0.99 (4)	C18—H18B	0.9600
C8—C9	1.369 (6)	C18—H18C	0.9600
C8—H8	0.88 (4)	C19—C20	1.510 (4)
C9—C10	1.371 (6)	C19—H19A	0.99 (3)
C9—H9	0.92 (4)	C19—H19B	1.02 (3)
C10—C11	1.387 (5)	C20—H20A	0.9600
C10—H10	0.86 (3)	C20—H20B	0.9600
C11—H11	0.91 (3)	C20—H20C	0.9600
N12—C13	1.452 (4)	C21—H21A	0.9600
N12—C14	1.455 (4)	C21—H21B	0.9600
C13—H13A	0.9600	C21—H21C	0.9600
C13—H13B	0.9600	C22—H22A	0.9600
C13—H13C	0.9600	C22—H22B	0.9600
C14—H14A	0.9600	C22—H22C	0.9600
			
C2—O1—C5	107.99 (17)	C3—C15—H15A	114.9 (16)
C3—C2—O1	110.01 (19)	N16—C15—H15A	104.0 (16)
C3—C2—C22	134.9 (2)	C3—C15—H15B	110.6 (14)
O1—C2—C22	115.1 (2)	N16—C15—H15B	105.0 (14)
C2—C3—C4	106.6 (2)	H15A—C15—H15B	107 (2)
C2—C3—C15	128.0 (2)	C21—N16—C19	109.63 (19)
C4—C3—C15	125.4 (2)	C21—N16—C17	106.23 (18)
C5—C4—N12	130.6 (2)	C19—N16—C17	113.2 (2)
C5—C4—C3	106.6 (2)	C21—N16—C15	109.68 (19)
N12—C4—C3	122.8 (2)	C19—N16—C15	109.27 (17)
C4—C5—O1	108.81 (19)	C17—N16—C15	108.73 (18)
C4—C5—C6	135.9 (2)	C18—C17—N16	115.0 (2)
O1—C5—C6	115.1 (2)	C18—C17—H17A	111.4 (15)
C11—C6—C7	119.0 (3)	N16—C17—H17A	104.1 (16)
C11—C6—C5	119.9 (2)	C18—C17—H17B	107.9 (17)
C7—C6—C5	121.1 (3)	N16—C17—H17B	105.2 (17)
C8—C7—C6	120.1 (3)	H17A—C17—H17B	113 (2)
C8—C7—H7	121 (2)	C17—C18—H18A	109.5
C6—C7—H7	118 (2)	C17—C18—H18B	109.5
C9—C8—C7	120.7 (3)	H18A—C18—H18B	109.5
C9—C8—H8	119 (3)	C17—C18—H18C	109.5
C7—C8—H8	120 (3)	H18A—C18—H18C	109.5
C10—C9—C8	119.6 (3)	H18B—C18—H18C	109.5
C10—C9—H9	118 (2)	C20—C19—N16	115.2 (2)
C8—C9—H9	123 (2)	C20—C19—H19A	112.9 (16)
C9—C10—C11	120.8 (4)	N16—C19—H19A	104.9 (17)
C9—C10—H10	124 (2)	C20—C19—H19B	109.8 (16)
C11—C10—H10	115 (2)	N16—C19—H19B	103.7 (16)
C10—C11—C6	119.8 (3)	H19A—C19—H19B	110 (2)
C10—C11—H11	119 (2)	C19—C20—H20A	109.5
C6—C11—H11	121 (2)	C19—C20—H20B	109.5
C4—N12—C13	116.8 (2)	H20A—C20—H20B	109.5
C4—N12—C14	114.6 (2)	C19—C20—H20C	109.5
C13—N12—C14	113.9 (3)	H20A—C20—H20C	109.5
N12—C13—H13A	109.5	H20B—C20—H20C	109.5
N12—C13—H13B	109.5	N16—C21—H21A	109.5
H13A—C13—H13B	109.5	N16—C21—H21B	109.5
N12—C13—H13C	109.5	H21A—C21—H21B	109.5
H13A—C13—H13C	109.5	N16—C21—H21C	109.5
H13B—C13—H13C	109.5	H21A—C21—H21C	109.5
N12—C14—H14A	109.5	H21B—C21—H21C	109.5
N12—C14—H14B	109.5	C2—C22—H22A	109.5
H14A—C14—H14B	109.5	C2—C22—H22B	109.5
N12—C14—H14C	109.5	H22A—C22—H22B	109.5
H14A—C14—H14C	109.5	C2—C22—H22C	109.5
H14B—C14—H14C	109.5	H22A—C22—H22C	109.5
C3—C15—N16	114.28 (18)	H22B—C22—H22C	109.5
			
C5—O1—C2—C3	−1.5 (3)	C6—C7—C8—C9	0.7 (5)
C5—O1—C2—C22	178.2 (2)	C7—C8—C9—C10	−1.2 (6)
O1—C2—C3—C4	1.8 (3)	C8—C9—C10—C11	1.6 (6)
C22—C2—C3—C4	−177.9 (3)	C9—C10—C11—C6	−1.5 (5)
O1—C2—C3—C15	−176.7 (2)	C7—C6—C11—C10	1.0 (4)
C22—C2—C3—C15	3.7 (4)	C5—C6—C11—C10	179.8 (3)
C2—C3—C4—C5	−1.4 (3)	C5—C4—N12—C13	−43.1 (4)
C15—C3—C4—C5	177.2 (2)	C3—C4—N12—C13	134.8 (2)
C2—C3—C4—N12	−179.7 (2)	C5—C4—N12—C14	93.7 (3)
C15—C3—C4—N12	−1.2 (4)	C3—C4—N12—C14	−88.3 (3)
N12—C4—C5—O1	178.6 (2)	C2—C3—C15—N16	86.9 (3)
C3—C4—C5—O1	0.5 (3)	C4—C3—C15—N16	−91.3 (3)
N12—C4—C5—C6	3.9 (5)	C3—C15—N16—C21	−67.4 (2)
C3—C4—C5—C6	−174.3 (3)	C3—C15—N16—C19	52.8 (3)
C2—O1—C5—C4	0.6 (3)	C3—C15—N16—C17	176.8 (2)
C2—O1—C5—C6	176.6 (2)	C21—N16—C17—C18	176.8 (2)
C4—C5—C6—C11	128.8 (3)	C19—N16—C17—C18	56.4 (3)
O1—C5—C6—C11	−45.7 (3)	C15—N16—C17—C18	−65.2 (3)
C4—C5—C6—C7	−52.4 (4)	C21—N16—C19—C20	−59.0 (3)
O1—C5—C6—C7	133.1 (3)	C17—N16—C19—C20	59.4 (3)
C11—C6—C7—C8	−0.5 (4)	C15—N16—C19—C20	−179.2 (2)
C5—C6—C7—C8	−179.4 (3)		
